# Risk Factors for Preterm Birth among HIV-Infected Tanzanian Women: A Prospective Study

**DOI:** 10.1155/2014/261689

**Published:** 2014-09-28

**Authors:** Rachel M. Zack, Jenna Golan, Said Aboud, Gernard Msamanga, Donna Spiegelman, Wafaie Fawzi

**Affiliations:** ^1^Department of Epidemiology, Harvard School of Public Health, 1633 Tremont Street, Boston, MA 02115, USA; ^2^Department of Nutrition, Harvard School of Public Health, Boston, MA, USA; ^3^Department of Microbiology and Immunology, Muhimbili University of Health and Allied Sciences, Dar es Salaam, Tanzania; ^4^Department of Community Health, Muhimbili University of Health and Allied Sciences, Dar es Salaam, Tanzania; ^5^Department of Biostatistics, Harvard School of Public Health, Boston, MA, USA; ^6^Department of Global Health and Population, Harvard School of Public Health, Boston, MA, USA

## Abstract

Premature delivery, a significant cause of child mortality and morbidity worldwide, is particularly prevalent in the developing world. As HIV is highly prevalent in much of sub-Saharan Africa, it is important to determine risk factors for prematurity among HIV-positive pregnancies. The aims of this study were to identify risk factors of preterm (<37 weeks) and very preterm (<34 weeks) birth among a cohort of 927 HIV positive women living in Dar es Salaam, Tanzania, who enrolled in the Tanzania Vitamin and HIV Infection Trial between 1995 and 1997. Multivariable relative risk regression models were used to determine the association of potential maternal risk factors with premature and very premature delivery. High rates of preterm (24%) and very preterm birth (9%) were found. Risk factors (adjusted RR (95% CI)) for preterm birth were mother <20 years (1.46 (1.10, 1.95)), maternal illiteracy (1.54 (1.10, 2.16)), malaria (1.42 (1.11, 1.81)), *Entamoeba coli* (1.49 (1.04, 2.15)), no or low pregnancy weight gain, and HIV disease stage ≥2 (1.41 (1.12, 1.50)). Interventions to reduce pregnancies in women under 20, prevent and treat malaria, reduce *Entamoeba coli* infection, and promote weight gain in pregnant women may have a protective effect on prematurity.

## 1. Introduction

Preterm delivery is recognized as a significant cause of child mortality and morbidity worldwide. Preterm birth is the leading cause of neonatal mortality [1]. It is estimated that preterm birth is the direct cause of 29% of deaths in children under 28 days and 11% of deaths in children under 5 years [1–3].

Prematurity disproportionately affects newborns in resource-constrained countries, where it is both more common and more often leads to adverse health outcomes. A systematic review from 2010 reports that 7.5% and 11.9% of births in developed countries and in Africa, respectively, were preterm [4]. In Tanzania, 11% of births are premature and prematurity is the second leading cause of neonatal death [5]. In a study of hospitalized neonates in Tanzania, the neonatal mortality rate for preterm infants was twice as high as that for full-term infants, 26% versus 13% [6].

Expending extra resources on additional medical care for pregnant women with risk factors for prematurity could reduce the incidence of prematurity. Not only would this have immediate benefits, such as reducing neonatal mortality, but it could also reduce the risk of chronic disease due to preterm delivery throughout the life course [7, 8]. This could substantially lower long-term health expenditures.

The prevalence of HIV infection among Tanzanian women is estimated to be 6.6% [9]. Given the significant proportion of pregnant women who are HIV-infected in some regions of the world, it is important to determine not only the risk factors for prematurity among the general population but also risk factors for prematurity among HIV-infected women, because such factors could be distinct from those in HIV uninfected women.

As the 2015 deadline of the Millennium Development Goals (MDGs) draws near, it is clear that MDG 4, the goal of reducing the under-five child mortality rate by two-thirds between 1990 and 2015, cannot be reached unless efforts are drastically increased [10]. While under-five mortality is declining in many countries, neonatal mortality still lags behind. Since preterm birth is responsible for 11% of under-five child mortality worldwide, knowledge of risk factors for prematurity may help the global community to reach MDG 4 [11]. This paper aims to contribute to the evidence base needed for a reduction in premature delivery by finding determinants of preterm and very preterm birth through the analysis of a cohort of 927 HIV-infected Tanzanian women. We examined potential risk factors for preterm delivery, many of which were previously found to be associated with preterm delivery in healthy populations.

## 2. Methods

### 2.1. Study Population and Design

Study participants were enrolled in a trial of vitamin supplements on pregnancy outcomes and HIV disease progression. Participants were HIV-infected pregnant women living in Dar es Salaam, Tanzania. Women were recruited from four prenatal clinics. They were enrolled in the study between April 1995 and July 1997. Inclusion criteria included 12 to 27 weeks gestation, intention to continue residing in Dar es Salaam for at least one year following delivery, World Health Organization (WHO) HIV disease stages 1–3, and informed consent to be randomized to a treatment regimen. The study has previously been described in further detail [12–15].

Of 1078 women enrolled in the trial, 949 had a live birth with known gestational age at delivery. For the prematurity analyses, we excluded 22 additional women because of missing data on key potential risk factors for preterm birth including age, maternal literacy, maternal malaria, HIV disease stage, hypertension status, height, or at least two measures of weight during pregnancy. None of the women reported smoking during pregnancy. This resulted in a sample of 927 pregnant women.

Consistent with the Tanzanian Ministry of Health's standard of prenatal care at the time of the study, study participants were provided with anemia and malaria prophylaxis during pregnancy. During pregnancy, all study participants received daily doses of 400 mg of ferrous sulfate and 5 mg of folic acid for anemia prophylaxis and weekly doses of 500 mg of chloroquine phosphate for malaria prophylaxis. Study participants were treated for hypertension and syphilis during pregnancy if found to have either disease. Study participants did not receive antiretrovirals (ARVs), as they were not available in Tanzania at the time of the study.

The trial was approved by the Research and Publications Committee of Muhimbili University of Health and Allied Sciences, the ethics committee of the Tanzania Ministry of Health's National AIDS Control Program, and the Institutional Review Board of the Harvard School of Public Health.

### 2.2. Measurements

Premature was defined as birth at fewer than 37 weeks of gestation and very premature was defined as fewer than 34 weeks of gestation. Gestational age was based on last menstrual period (LMP), which was self-reported by the participant at both screening and enrollment. Fundal height was measured as the distance, in centimeters, from the top of the pubic bone to the top of the uterus by trained doctors during the screening visit.

Anthropometric measurements, genital swabs, and blood, urine, and stool samples were collected at enrollment. These specimens were used to diagnose malaria, sexually transmitted infections (STIs), and parasitic infections and to measure micronutrient levels, hemoglobin concentration, HIV viral load, and CD4 cell count. By design, vitamin A, vitamin E, viral load, parasitic infections, and STIs were only measured in a subsample of study participants.

The following STIs were tested for at enrollment: trichomoniasis, gonorrhea, syphilis, and vaginal candidiasis. Syphilis was diagnosed as active if syphilis antibodies from sera were found to be present by both the Venereal Disease Research Laboratory (VDRL; Murex Diagnostic, Dartford, United Kingdom) and* Treponema pallidum* haemagglutination (TPHA; Fijurebio, Tokyo, Japan) tests. Gonorrhea was diagnosed based on a culture of the genital swab to detect* Neisseria gonorrhea* and* Trichomonas vaginalis* was diagnosed based upon wet mounts that were prepared and examined using microscopy. Vaginal candidiasis was diagnosed clinically by physician exam.

The following intestinal parasitic infections were tested for at enrollment:* Ascaris lumbricoides*,* Cryptosporidium parvum*,* Entamoeba coli*,* Entamoeba histolytica*,* Enterobius*,* Giardia lamblia*, hookworm, isospora, microsporidia,* Stronglyoides stercoralis*,* Taenia,* and* Trichuris trichiura*. Stool samples were examined macroscopically for the previously mentioned worms and microscopically using saline and iodine wet mount and the formalin-ether concentration technique for larvae, ova, and cysts.

Malaria was diagnosed by use of thick-smear blood films stained with Giemsa. Vitamin A deficiency was defined as plasma vitamin A <20 *μ*g/dL. Low vitamin E levels were defined as plasma vitamin E <9.7 *μ*mol/L. CD4 cell counts were dichotomized by <200 cell/mm^3^. We categorized hemoglobin levels according to three cutpoints: 7.0, 8.5, and 11.0 g/L. The WHO defines anemia in pregnancy as a hemoglobin concentration below 11 g/dL [16]. Prehypertension was defined as systolic blood pressure (SBP) ≥120 or diastolic blood pressure (DBP) ≥80 and hypertension was defined as SBP ≥140 or DBP ≥90 [17]. Selenium and mid-upper arm circumference (MUAC) were categorized by quartile. WHO HIV disease stage was dichotomized (stage < 2 versus stage ≥ 2) because of the limited number of women with HIV stage >2.

Trained research nurses interviewed study participants at enrollment. Maternal age and relationship status were self-reported. Maternal age at conception was estimated by subtracting gestational age at enrollment from maternal age at enrollment. Having a partner was defined as self-report of being married or cohabiting.

### 2.3. Statistical Analyses

Prematurity status, the outcome variable, was determined based on self-reported LMP. In order to validate the quality of LMP as a measure of gestational age we performed a supplemental analysis where we calculated the Spearman correlation between gestational ages based on LMP and fundal height.

Risk factors measured at enrollment that we examined were mother's age at conception, having no partner, being illiterate, mid-upper arm circumference (MUAC), height, vitamin A, vitamin E, selenium, hemoglobin, malaria, parasitic infections, STIs, HIV disease stage ≥ 2, CD4 cell count, and viral load. Whether or not the pregnancy resulted in twins and weight change during pregnancy were also included as exposures. Although the Tanzania Vitamin and HIV Infection Trial found that multivitamins reduced the risk of very preterm delivery [12], we did not adjust for assigned vitamin regimen because vitamin regimen was randomized and thus did not confound the relationships between the risk factors we examined and preterm delivery.

Weight change during pregnancy was calculated for each individual by using linear regression to regress weight on week of gestation [18–20]. Weight change was then categorized into three groups: no gain (≤0 grams per week), gain below the 25th percentile, and gain at or above the 25th percentile.

Missing indicators were created for each of the parasitic infections, each of the STIs, viral load, CD4 count, vitamin A deficiency, vitamin E, selenium, hemoglobin, and MUAC [21].

Unadjusted and adjusted relative risk regression models were run to calculate relative risks (RRs) and 95% confidence intervals (CIs) [22]. Log-binomial regression was used for all unadjusted regressions. Poisson was used for the adjusted regressions because the adjusted log-binomial regressions did not converge. Each regression was run twice, once with preterm versus full term as the dependent variable and again with very preterm versus full term as the dependent variable. Deliveries of ≥34–<37 weeks were excluded from the very preterm versus full term analysis. Explanatory variables were included in the adjusted models if the *P*-value in the unadjusted model was less than 0.2 [23]. Wald tests for trend were calculated for categorical variables by taking the median value of each category and setting missing values to the overall median. Wald tests were calculated for dichotomous and continuous variables.

We calculated a Spearman correlation coefficient comparing gestational age as measured by LMP and fundal height on 1077 women. The analysis included data on all trial participants with a known LMP and fundal height at the screening visit, excluding only one woman who was missing data on LMP.

A *P* value of ≤0.05 was considered to be statistically significant. All statistical analyses were performed using SAS software 9.3 (SAS Institute, Inc., http://www.sas.com, Cary, North Carolina).

## 3. Results

The median gestational age at enrollment was 21 weeks (IQR 18–23). The median gestational age at delivery was 39 weeks (IQR 37–41). Seventy-six percent of deliveries were full-term, 24% were premature and 9% were very premature. The characteristics of the cohort are shown in Table 1.

The results of unadjusted and adjusted relative risk regressions assessing the relationships of maternal factors and the risk for preterm versus full-term deliveries are shown in Table 2. After adjustment, maternal age less than 20 years (RR, 1.46; 95% CI, 1.10–1.95), maternal illiteracy (RR, 1.54; 95% CI, 1.10–2.61), low weight gain during pregnancy (*P* for trend = 0.006), malaria (RR, 1.42; 95% CI, 1.11–1.81),* Entamoeba coli* infection (RR, 1.49; 95% CI, 1.04–2.15), and HIV disease stage ≥ 2 (RR, 1.41; 95% CI, 1.12–1.79), and prehypertension or hypertension (RR, 1.26; 95% CI, 1.00–1.59) were significantly and independently associated with increased risk of preterm delivery. After adjustment, vitamin A deficiency,* Entamoeba histolytica* infection, and carrying twins were all associated with an increased risk of preterm delivery with borderline significance.

The results of unadjusted and adjusted relative risk regressions comparing maternal factors for very preterm versus full-term deliveries are presented in Table 3. Maternal age less than 20 years (RR, 1.91; 95% CI, 1.21–3.03), small MUAC (*P* for trend = 0.03), low weight gain during pregnancy (*P* for trend = 0.0005), malaria (RR, 1.81; 95% CI, 1.19–2.76),* Entamoeba coli* infection (RR, 2.38; 95% CI, 1.38–4.09), and* Entamoeba histolytica* infection (RR, 2.62; 95% CI, 1.09–6.28), and HIV disease stage ≥ 2 (RR, 1.68; 95% CI, 1.11–2.53) were significantly and independently associated with increased risk of preterm delivery. Maternal illiteracy was associated with increased risk of very preterm delivery with borderline significance.

For the analysis, we dichotomized maternal age with a cutpoint at 20 years. We chose not to include an age group for older mothers because analyses showed that the risk of prematurity in mothers aged 30 and older was not significantly greater than the risk of prematurity in mothers aged 20 to 30 (data not shown).

There may be concern that gestational age based on self-reported LMP is potentially unreliable. However, we found a correlation of 0.78 (95% CI, 0.76–0.80) between gestational age at screening based on LMP and fundal height, suggesting that, for this population, self-reported LMP can be used as a reasonable measure of gestational age.

## 4. Discussion

### 4.1. Main Findings

Our study found that HIV disease stage ≥ 2, no or low weight gain during pregnancy,* Entamoeba coli*, malaria, and maternal age less than 20 years were significantly associated with risk of preterm and very preterm delivery. Illiteracy and pre-hypertension/hypertension were associated with preterm delivery; however, the relationship of illiteracy with very preterm was only borderline significant and pre-hypertension/hypertension was not associated with very preterm delivery.* Entamoeba histolytica*, an intestinal parasitic infection, and MUAC were associated with very preterm delivery but not preterm delivery.

### 4.2. Strengths and Limitations

Although there have been several studies on risk factors for premature delivery, this study is unique in that it is based upon a large sample of HIV-infected, ART-naïve pregnant women in sub-Saharan Africa. However, there are some potential limitations to our study and analyses. Many of the potential risk factors we examined were only measured at enrollment and not throughout the pregnancy. Additional useful information would have been obtained if lab samples had been taking throughout participants' pregnancies, since this would have provided more complete data on parasitic infections, STIs, HIV disease progression, and nutritional status throughout pregnancy.

The potential lack of reliability of using LMP to estimate gestational age is a limitation to our study, although this approach is used frequently, both clinically and for research, in sub-Saharan Africa given the expense and lack of access to ultrasound machines. We expect that the definition of prematurity will be misclassified in a random way with respect to various strata of a particular risk factor in our analyses. Thus, we expect that our findings are biased towards the null because nondifferential exposure of a binary outcome will bias the results towards the null [24]. We found gestational age based on fundal height and LMP to be highly correlated. However, ultrasound, not fundal height measurements is the gold standard for measuring gestational age. Fundal height is thought to be accurate to 1–3 weeks. A future study comparing gestational age based on LMP versus ultrasound in a developing country context, where recall of LMP may be especially inaccurate, could provide further information on the reliability of gestational age based on self-reported LMP.

Our study took place before zidovudine (AZT) and other ARVs were in use in Tanzania, allowing us to identify risk factors for prematurity among children born to ARV-naive HIV-positive women. The use of highly active antiretroviral therapy (HAART), but not AZT monotherapy, during pregnancy has been shown to increase the risk of preterm delivery [25]. In 2012, only 71% of HIV-infected pregnant women in Tanzania were receiving ARVs for prevention of mother-to-child transmission (PMTCT) [26]. This indicates that in Tanzania the risk factors we have found for preterm delivery in HIV-infected women are directly relevant for 29% of the pregnancies in HIV-infected women. A review of PMTCT coverage in 108 countries between 2007 and 2009 found that only 35% of HIV-infected pregnant women received PMCTC [27]. The WHO estimates that in 21 priority countries in sub-Saharan Africa in 2012, only 64% of HIV-infected pregnant women received any ARVs for PMCTC [28]. Among these 21 countries, PMCTC coverage ranged from 13% in the Democratic Republic of the Congo to >95% in both Botswana and Zambia [28].

Furthermore, the WHO guidelines recommend that low-income countries choose from one of three options (A, B, or B+) for PMTCT. The guidelines suggest that all patients with a CD4 count of <350 cells/mm^3^ begin HAART and that pregnant women with CD4 counts >350 cells/mm^3^ be provided with either option A, B, or B+. Option A is the provision of AZT monotherapy beginning at no sooner than 14 weeks gestation. Option B is the provision of HAART beginning at no sooner than 14 weeks gestation. Option B+ is the provision of HAART as soon as HIV infection is diagnosed. While some countries have adopted option B or B+ for prevention of mother-to-child transmission of HIV, several countries have opted to adopt Option A of the WHO guidelines whereby women receive HAART only if they are advanced in their disease. For example, Kenya has adopted a combination of options A and B, in which health centers adopt option A if they do not have the capacity to adopt option B [29]. In these settings, a majority of pregnant women are in early stages of disease and are thus not initiated on triple ARVs, and for whom our findings are applied.

### 4.3. Interpretation

Preterm delivery and very preterm delivery were analyzed as separate outcomes for two reasons. First, because very preterm delivery leads to more severe health outcomes than preterm delivery, identification of differing risk factors would make possible the targeting of available resources toward very preterm delivery prevention. However, we found that risk factors for very preterm and preterm deliveries were similar. Our results suggest that the same interventions can be used to reduce both preterm and very preterm deliveries. Furthermore, analyzing preterm and very preterm deliveries separately provided a sensitivity analysis. The regression analysis of very preterm deliveries excluded deliveries between 34 and 37 weeks, reducing misclassification bias that may be associated with gestational age assessment.

Because very preterm deliveries are a subset of preterm deliveries, they have a smaller sample size, resulting in larger standard errors and *P* values, and wider confidence intervals. Many of the point estimates are larger in the very preterm regression. This may be because there is less misclassification bias due to incorrectly estimated gestational age. Deliveries with a reported gestational age of less than 34 weeks were more likely to be correctly classified as preterm than those with a reported gestational age of less than 37 weeks.

The rate of prematurity that we observed, 24%, is higher than that found in other HIV-infected populations. A study of 1626 HIV-positive Nigerian women from 2004–2010 found a preterm delivery rate of 11.1% [30]. This may partially be due to improvements in nutrition and maternal health from 1995–1997 to 2004–2010. A study in the United States in the early 1990's found that 19% of infants born to HIV-infected mothers were premature [31]. This difference is likely due, in part, to better healthcare in the US.

We found lack of weight gain during pregnancy to be associated with preterm and very preterm delivery. Weight gain is a marker of maternal nutritional status, and thus no or low weight gain during pregnancy could be a marker of inadequate nutrition during pregnancy. However, this finding may be due to reverse causality, as it is unknown whether lack of weight gain caused the premature delivery or the premature delivery led to less time for gestational weight gain.

We found that HIV disease stage ≥ 2 at enrollment increased the risk of prematurity. However, we did not find any further association between prematurity and either CD4 cell count or viral load. A study in South Africa found no effect of HIV-infection in ART-naïve women on prematurity [32]. However, a previous study in China did report an effect of CD4 count and viral load on prematurity [33].

We found younger maternal age, but not older maternal age, to be a risk factor for preterm birth. Women younger than 20 years were more likely to deliver preterm compared to women 20 years and older. However, women 30 years of age and older were not found to be at a higher risk of premature delivery compared to women aged 20–30. The result that young mothers are at an increased risk for premature delivery has also been found in studies in other sub-Saharan African countries such as Cameroon [34], but not Zimbabwe [35]. The result that older maternal age is not associated with premature delivery was also found in a Turkish study that found adolescent pregnancy to be a weak risk factor for prematurity but did not find maternal age of 39 and older to increase the risk of prematurity [36]. However, a study in the US found women aged 30–34 and aged 35 and older to be at an increased risk for premature delivery compared to women aged 25–29 [37].

## 5. Conclusion

As the preventable and treatable major killers of children such as malaria, diarrheal disease, and respiratory disease are increasingly managed, with the presumption that mortality from these causes will decrease, the proportion of child mortality due to premature birth will increase. In order to further reduce needless child deaths, it will be important to reduce the modifiable determinants of preterm birth so as to increase the percentage of children that are born full-term. Interventions to promote nutritional status and to slow HIV disease progression are likely to be critical for reducing the risk of prematurity.

## Figures and Tables

**Table 1:** Study population characteristics, *N* = 927^a^.

Characteristic	*N* (%) or median [IQR]
Preterm births (<37 wks)	227 (24%)
Very preterm births (<34 wks)	87 (9%)
Gestational age at enrollment (weeks)	21 [18, 23]
Gestational age at birth (weeks)	39 [37, 41]
Sociodemographic	
Maternal age at conception (years)	25 [22, 29]
<20	96 (10%)
No partner	103 (11%)
Illiterate	70 (8%)
Nutrition and Anthropometric	
Plasma vitamin A (*µ*g/dL)	23.4 [17.9, 30.2]
<20	246 (34%)
Plasma vitamin E (*µ*mol/L)	9.6 [8.0, 11.4]
<9.7	368 (51%)
Selenium (mg/mL)	0.12 [0.11, 0.14]
<0.11	218 (26.49%)
0.11-0.12	148 (17.98%)
0.12–0.14	250 (30.38%)
≥0.14	207 (25.15%)
Hemoglobin (g/dL)	9.5 [8.4, 10.5]
<7.0	59 (6%)
7.0–8.4	188 (21%)
8.5–10.9	508 (56%)
≥11.0	112 (12%)
Mid-upper arm circumference (cm)	25.0 [23.5, 27.5]
≤23.5	225 (26%)
23.6–25.0	203 (24%)
25.1–27.5	220 (26%)
>27.5	203 (24%)
Weight gain (g per week)	255 [116, 380]
≤0	112 (12%)
1–119	126 (14%)
≥120	689 (74%)
Height (cm)	156 [152, 160]
<150	90 (10%)
Infections	
Malaria	176 (19%)
Hookworm	92 (12%)
*Entamoeba coli *	69 (9%)
*Ascaris lumbricoides *	43 (6%)
*Cryptosporidium parvum *	34 (4%)
*Entamoeba histolytica *	15 (2%)
*Strongyloides stercoralis *	14 (2%)
*Trichuris trichiura *	9 (1%)
*Giardia lamblia *	4 (1%)
*Enterobius *	0 (0%)
*Isospora *	0 (0%)
Microsporidia	0 (0%)
Taenia	0 (0%)
Trichomoniasis	232 (25%)
Syphilis	44 (6%)
Vaginal candidiasis	41 (6%)
Gonorrhea	9 (1%)
HIV/AIDS	
WHO HIV disease stage ≥ 2	189 (20%)
CD4 count (cells/mm^3^)	402 [275, 530]
<200	112 (13%)
Viral load (copies/mL)	47819 [11626, 146135]
≥50,000	193 (50%)
Other clinical variables	
Twins	22 (2%)
Diastolic blood pressure (mmHg)	70 [60, 70]
Systolic blood pressure (mmHg)	110 [100, 110]
Prehypertension or hypertension^b^	259 (28%)

AIQR: interquartile range.

^a^Not all numbers add up to 927 due to missing data.

^b^Systolic blood pressure > 120 mmHg or diastolic blood pressure > 80 mmHg.

**Table 2:** Risk factors for preterm birth (<37 weeks versus ≥37 weeks), *N* = 927.

Characteristic	Unadjusted		Adjusted^a^	
	RR [95% CI]	*P* value	RR [95% CI]	*P* value
Sociodemographic				
Age < 20 at conception	1.58 [1.18, 2.11]	0.002	1.47 [1.10, 1.97]	0.01
No partner	1.17 [0.84, 1.63]	0.35		
Illiterate	1.58 [1.14, 2.20]	0.006	1.53 [1.09, 2.15]	0.01
Nutrition				
Plasma vitamin A < 20 (*µ*g/dL)	1.24 [1.19, 1.96]	0.0008	1.25 [0.97, 1.62]	0.08
Plasma vitamin E < 9.7 (*µ*mol/L)	0.88 [0.69, 1.13]	0.31		
Selenium (mg/mL)		0.12		0.16
<0.11	1.37 [0.97, 1.96]		1.40 [0.98, 2.00]	
0.11-0.12	1.15 [0.77, 1.73]		1.11 [0.74, 1.65]	
0.12–0.14	1.39 [0.98, 1.95]		1.48 [1.05, 2.08]	
≥0.14	Reference		Reference	
Hemoglobin (g/dL)		0.11		0.90
<7.0	1.29 [0.83, 2.01]		0.98 [0.64, 1.52]	
7.0–8.4	1.05 [0.74, 1.49]		0.90 [0.63, 1.28]	
8.5–10.9	0.82 [0.60, 1.11]		0.78 [0.58, 1.07]	
≥11.0	Reference		Reference	
Mid-upper arm circumference (cm)		0.19		0.23
≤23.5	1.29 [0.92, 1.81]		1.32 [0.93, 1.87]	
23.6–25.0	0.95 [0.66, 1.39]		0.91 [0.63, 1.33]	
25.1–27.5	1.11 [0.78, 1.58]		1.07 [0.75, 1.53]	
>27.5	Reference		Reference	
Weight gain (g per week)		<0.0001		0.006
≤0	1.95 [1.51, 2.53]		1.60 [1.20, 2.14]	
1–120	1.18 [0.85, 1.65]		1.12 [0.81, 1.57]	
≥120	Reference		Reference	
Height < 150 cm	0.95 [0.64, 1.40]	0.79		
Infections				
Malaria	1.53 [1.20, 1.96]	0.0006	1.41 [1.10, 1.81]	0.006
Hookworm	1.30 [0.92, 1.84]	0.13	1.19 [0.83, 1.70]	0.34
*Entamoeba coli *	1.49 [1.04, 2.14]	0.03	1.49 [1.04, 2.15]	0.03
*Ascaris lumbricoides *	1.21 [0.73, 1.99]	0.46		
*Cryptosporidium parvum *	0.88 [0.45, 1.72]	0.70		
*Entamoeba histolytica *	1.73 [0.92, 3.27]	0.09	1.79 [0.99, 3.26]	0.06
*Strongyloides stercoralis *	1.23 [0.53, 2.84]	0.63		
*Trichuris trichiura *	1.44 [0.56, 3.65]	0.45		
Trichomoniasis	1.18 [0.92, 1.52]	0.19	1.13 [0.88, 1.45]	0.35
Syphilis	1.28 [0.80, 2.05]	0.31		
Vaginal candidiasis	1.10 [0.66, 1.85]	0.72		
Gonorrhea	1.83 [0.88, 3.85]	0.11	1.68 [0.84, 3.35]	0.14
HIV/AIDS				
WHO HIV disease stage ≥ 2	1.60 [1.26, 2.03]	0.0001	1.40 [1.11, 1.78]	0.005
CD4 count < 200 cells/mm^3^	0.92 [0.64, 1.32]	0.65		
Viral load ≥ 50,000 copies/mL	1.02 [0.74, 1.42]	0.89		
Other clinical variables				
Twins	1.50 [0.85, 2.64]	0.16	1.85 [0.94, 3.66]	0.07
Prehypertension or hypertension^b^	1.25 [0.98, 1.58]	0.07	1.27 [1.01, 1.60]	0.04

ARR: risk ratio; CI: confidence interval.

^a^Adjusted for age < 20 years, literacy, plasma vitamin A < 20 *µ*g/dL, selenium, mid-upper arm circumference, weight gain, malaria, hookworm, *Entamoeba coli*, *Entamoeba histolytica*, trichomoniasis, gonorrhea, WHO HIV disease stage ≥ 2, twins, and prehypertension or hypertension.

^b^Systolic blood pressure >120 mmHg or diastolic blood pressure >80 mmHg.

**Table 3:** Risk factors for very preterm birth (<34 weeks versus ≥37 weeks), *N* = 787.

Characteristic	Unadjusted		Adjusted^a^	
	RR [95% CI]	*P* value	RR [95% CI]	*P* value
Sociodemographic				
Age < 20 at conception	2.21 [1.37, 3.55]	0.001	1.94 [1.22, 3.06]	0.005
No partner	1.20 [0.66, 2.16]	0.55		
Illiterate	1.93 [1.09, 3.41]	0.02	1.66 [0.91, 3.02]	0.10
Nutrition				
Plasma vitamin A < 20 (*µ*g/dL)	1.89 [1.24, 2.87]	0.003	1.38 [0.89, 2.13]	0.15
Plasma vitamin E < 9.7 (*µ*mol/L)	0.86 [0.57, 1.32]	0.50		
Selenium (mg/mL)	0.003 [<0.01, 58]	0.24		
<0.11	1.64 [0.87, 3.09]			
0.11-0.12	1.13 [0.53, 2.40]			
0.12–0.14	1.82 [1.00, 3.32]			
≥0.14	Reference			
Hemoglobin (g/dL)		0.45		
<7.0	0.72 [0.43, 1.23]			
7.0–8.4	0.72 [0.43, 1.23]			
8.5–10.9	1.12 [0.63, 2.01]			
≥11.0	Reference			
Mid-upper arm circumference (cm)		0.03		0.03
≤23.5	1.90 [0.99, 3.67]		2.07 [1.03, 4.16]	
23.6–25.0	1.50 [0.75, 3.00]		1.50 [0.74, 3.06]	
25.1–27.5	1.10 [0.52, 2.32]		1.03 [0.49, 2.20]	
>27.5	Reference		Reference	
Weight gain (g per week)		<0.0001		0.0005
≤0	3.21 [2.06, 5.00]		2.32 [1.36, 3.96]	
1–120	1.78 [1.05, 3.03]		1.85 [1.05, 3.25]	
≥120	Reference		Reference	
Height < 150 cm	0.93 [0.47, 1.86]	0.84		
Infections				
Malaria	2.03 [1.34, 3.07]	0.0009	1.87 [1.20, 2.89]	0.005
Hookworm	1.18 [0.61, 2.27]	0.63		
*Entamoeba coli *	2.34 [1.36, 4.02]	0.002	2.38 [1.38, 4.09]	0.002
*Ascaris lumbricoides *	1.34 [0.58, 3.13]	0.49		
*Cryptosporidium parvum *	0.64 [0.17, 2.50]	0.53		
*Entamoeba histolytica *	2.44 [0.89, 6.66]	0.08	2.62 [1.09, 6.28]	0.03
*Strongyloides stercoralis *	0.86 [0.13, 5.65]	0.88		
*Trichuris trichiura *	No data			
Trichomoniasis	1.30 [0.84, 1.99]	0.24		
Syphilis	1.34 [0.58, 3.13]	0.49		
Vaginal candidiasis	0.82 [0.27, 2.45]	0.72		
Gonorrhea	2.61 [0.80, 8.57]	0.11	2.72 [0.86, 8.58]	0.09
HIV/AIDS				
WHO HIV disease stage ≥ 2	2.09 [1.39, 3.14]	0.0004	1.67 [1.11, 2.51]	0.01
CD4 count < 200 cells/mm^3^	0.72 [0.36, 1.45]	0.36		
Viral load ≥ 50,000 copies/mL	0.79 [0.46, 1.36]	0.40		
Other clinical variables				
Twins	2.06 [0.85, 5.00]	0.11	1.86 [0.68, 5.07]	0.22
Prehypertension or hypertension^b^	1.16 [0.76, 1.79]	0.49	1.22 [0.81, 1.85]	0.33

ARR: risk ratio; CI: confidence interval.

^a^Adjusted for age < 20 years, literacy, plasma vitamin A < 20 *µ*g/dL, mid-upper arm circumference, weight gain, malaria, *Entamoeba coli*, *Entamoeba histolytica*, gonorrhea, WHO HIV disease stage ≥ 2, twins, and prehypertension or hypertension.

^b^Systolic blood pressure > 120 mmHg or diastolic blood pressure > 80 mmHg.
